# Modelling the Transmission Dynamics of COVID-19 in Six High-Burden Countries

**DOI:** 10.1155/2021/5089184

**Published:** 2021-05-27

**Authors:** Azizur Rahman, Md Abdul Kuddus

**Affiliations:** ^1^Data Science Research Unit, School of Computing and Mathematics, Charles Sturt University, Wagga Wagga, NSW 2678, Australia; ^2^Australian Institute of Tropical Health and Medicine, James Cook University, Townsville, QLD 4810, Australia; ^3^Department of Mathematics, University of Rajshahi, Rajshahi 6205, Bangladesh

## Abstract

The new Coronavirus Disease 19, officially known as COVID-19, originated in China in 2019 and has since spread worldwide. We presented an age-structured Susceptible-Latent-Mild-Critical-Removed (SLMCR) compartmental model of COVID-19 disease transmission with nonlinear incidence during the pandemic period. We provided the model calibration to estimate parameters with day-wise COVID-19 data, i.e., reported cases by worldometer from 15^th^ February to 30^th^ March 2020 in six high-burden countries, including Australia, Italy, Spain, the USA, the UK, and Canada. We estimate transmission rates for each country and found that the country with the highest transmission rate is Spain, which may increase the new cases and deaths than the other countries. We found that saturation infection negatively impacted the dynamics of COVID-19 cases in all the six high-burden countries. The study used a sensitivity analysis to identify the most critical parameters through the partial rank correlation coefficient method. We found that the transmission rate of COVID-19 had the most significant influence on prevalence. The prediction of new cases in COVID-19 until 30^th^ April 2020 using the developed model was also provided with recommendations to control strategies of COVID-19. We also found that adults are more susceptible to infection than both children and older people in all six countries. However, in Italy, Spain, the UK, and Canada, older people show more susceptibility to infection than children, opposite to the case in Australia and the USA. The information generated from this study would be helpful to the decision-makers of various organisations across the world, including the Ministry of Health in Australia, Italy, Spain, the USA, the UK, and Canada, to control COVID-19.

## 1. Introduction

**Figure 1:**
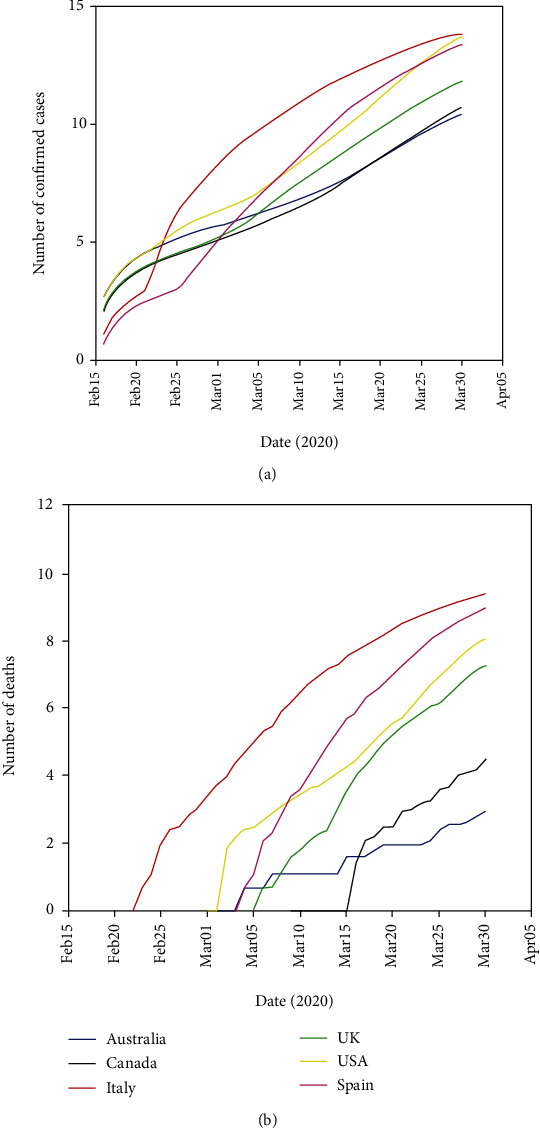
Graphs of six selected countries using a log scale: (a) cumulative number of COVID-19 cases and (b) cumulative number of COVID-19 deaths.

Following the outbreak of the novel Severe Acute Respiratory Syndrome Coronavirus-2 (SARS-CoV-2), COVID-19 constitutes a persistent and significant public health problem worldwide. As of 30^th^ March 2020, the ongoing global pandemic outbreak of COVID-19 has spread to at least 180 countries and territories, including Australia, Italy, Spain, the USA, the UK, and Canada, and resulted in approximately 946,876 cases of COVID-19 and 48,137 deaths [1]. In Australia, Italy, Spain, the USA, the UK, and Canada, COVID-19 infections and deaths reached 4460, 101739, 87956, 163788, 22141, and 7448, as well as 30, 11591, 7716, 3143, 1408, and 89, with mortality ratios of nearly 0.67%, 11.39%, 8.77%, 1.9%, 6.4%, and 1.2%, respectively [1].  Figure 1 shows the cumulative number of confirmed cases and deaths of COVID-19 in six selected countries from 15^th^ February to 30^th^ March 2020.

The highest burden of COVID-19 is reliant on the health system and depends on a quick and timely response to the pandemic. For example, in Italy, the first confirmed COVID-19 cases were on February 15, and then, after a few days, thousands of people were infected by COVID-19. The problem is not that the Italian government did not respond to the COVID-19. The problem is that it always responded slightly too slow and with slightly too much moderation. What has resulted in China reveals that quarantine, social distancing, and isolation of infected populations can contain the pandemic. This impact of the COVID-19 response in China is being advocated in many countries where COVID-19 is starting to spread. However, it is unclear whether other countries can implement the stringent measures China eventually adopted. Singapore and Hong Kong, both of which had severe acute respiratory syndrome (SARS) epidemics in 2002–2003, present concern and many lessons to other countries. In both places, COVID-19 has been maintained well to date, notwithstanding early cases, by early government progress and through social distancing patterns used by individuals.

A series of critical factors can lead to the outbreak of the COVID-19 pandemic. However, some of those factors seem to be poorly understood. Mathematical modelling is a powerful tool for infectious disease control that helps to accurately predict behaviour and understand infectious disease dynamics [2–4]. Many researchers have implemented mathematical modelling frameworks to gain insights into different infectious diseases [5–9]. Although models can range from very simple to highly complex, one of the most typical practices to improve understanding of infectious disease dynamics is the compartmental mathematical model [10].

In mathematical models, the incidence rate plays a vital role in the transmission of infectious diseases. The number of individuals who become infected per unit time is called the incidence rate in the epidemiology perspective [11]. Here, we consider the nonlinear incidence rate because the number of effective contacts between infective and susceptible individuals may saturate at high levels through the crowding of infective individuals [12]. This model is also used to calibrate and predict the number of COVID-19 case data in six countries, including Australia, Italy, Spain, the USA, the UK, and Canada, to estimate the model parameters. We assessed the impact of age structure on the dynamics of COVID-19 cases in all six burden countries. The study performed a sensitivity analysis to identify the essential model parameters that could support policymakers in controlling the COVID-19 outbreak in the selected countries. The model findings can be also helpful to many other countries which are dealing with the critical outbreak of COVID-19.

The rest of the paper is structured as follows: Section 2 presents model descriptions. Sections 3, 4, and 5, respectively, performed the model calibration and impact of saturation infection, the impact of age structure, and sensitivity analysis.  Section 6 finalizes the paper with a brief discussion and concluding remarks.

## 2. Model Description and Analysis

**Figure 2:**
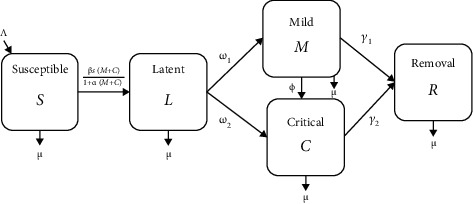
Flow chart of the SLMCR mathematical model showing the five states and the transitions in and out of each state. *S*: susceptible population; *L*: latent population (not yet symptomatic); *M*: mild population (moderate symptom); *C*: critical population (severe symptom); *R*: removal population; *Λ*: recruited rate; *μ*: death rate; *β*: transmission rate; *α*: force of saturation infection; *ω*_1_: progression rate from latent to a mild compartment; *ω*_2_: progression rate from the latent critical compartment; *γ*_1_: recovery rate from mild to removal compartment; *γ*_2_: recovery rate from critical to removal compartment; *ϕ*: progression rate from mild to critical compartment due to the comorbidities with other diseases.

We considered an SLMCR compartmental model with three age classes: children (0-14 years), adults (15-64 years), and older (over 64 years) of COVID-19 transmission with a nonlinear incidence between the following mutually exclusive compartments: *S*(*t*): susceptible individuals; *L*(*t*): latent individuals, representing those who are infected and have not yet developed active COVID-19; *M*(*t*): mild individuals who are both infected and infectious and have mild respiratory illness symptoms such as nasal congestion, runny nose, and a sore throat; *C*(*t*): critical individuals who are both infected and infectious and have severe symptoms including shortness of breath, chest discomfort, and bluish face; and *R*(*t*): recovered individuals who are previously infected but successfully recovered.  Figure 2 depicts a typical SLMCR model.

Let the susceptible individuals be recruited at a constant rate *Λ*, and they may be infected at a time-dependent rate *β*(*M* + *C*)/(1 + *α*(*M* + *C*)). Here, *β*(*M* + *C*)/(1 + *α*(*M* + *C*)) represents the saturated incidence rate, which tends to a saturated level when (*M* + *C*) gets large. *β*(*M* + *C*) measures the force of infection when the disease is entering a fully susceptible population, and 1/(1 + *α*(*M* + *C*)) measures the inhibition effect from the behaviour change of susceptible individuals when their number increases or from the effect of risk factors including a crowded environment of the infective individuals with *α* which determines the level at which the force of infection saturates. Individuals in different compartments suffer from natural death at the same constant rate *μ*. All infected individuals move to the latently infected compartment, *L*(*t*). Those with latent infection progress to mild and critical infections (the *M* and *C* compartments) due to reactivation of the latent infection at rate *ω*_1_ and *ω*_2_, respectively. However, some mild populations also move to the critical compartment due to the comorbidities with other diseases, including hypertension, diabetes, cardiovascular disease, and respiratory system disease [13]. A proportion of the mild and critical individuals recover through treatment and natural recovery rates *γ*_1_ and *γ*_2_, respectively, and move into the recovered compartment *R*(*t*). In this case, we can express the model by the following five differential equations with an age-structured contact matrix, P:

(1) $$\begin{array}{c} \frac{{\text{d}S}_{i}}{\text{d}t}=\Lambda -\frac{\beta S_{i}}{1+\alpha \left(M_{i}+C_{i}\right)}\;\underset{j}{\sum }{\text{P}}_{ij}\left(M_{j}+C_{j}\right)-\mu S_{i}, \end{array}$$

(2) $$\begin{array}{c} \frac{\text{d}L_{i}}{\text{d}t}=\frac{\beta S_{i}}{1+\alpha \left(M_{i}+C_{i}\right)}\;\underset{j}{\sum }P_{ij}\left(M_{j}+C_{j}\right)-\left(\omega_{1}+\omega_{2}+\mu \right)L_{i}, \end{array}$$

(3) $$\begin{array}{c} \frac{{\text{d}M}_{i}}{\text{d}t}=\omega_{1}L_{i}-\left(\phi +\gamma_{1}+\mu \right)M_{i}, \end{array}$$

(4) $$\begin{array}{c} \frac{{\text{d}C}_{i}}{\text{d}t}=\omega_{2}L_{i}+\phi M_{i}-\left(\gamma_{2}+\mu \right)C_{i}, \end{array}$$

(5) $$\begin{array}{c} \frac{{\text{d}R}_{i}}{\text{d}t}=\gamma_{1}M_{i}+\gamma_{2}C_{i}-\mu R_{i}, \end{array}$$

where *i* and *j* are the indices of the age classes and P represents the contact matrix. The matrix P has satisfied reciprocity, meaning that within the population, the total time spent by the children with adults and older people, the adults with children and older people, and the older people with children and adults must be equal to the time spent by the adults and older people with children, the children and older people with adults, and the adults and children with older people.

Given the nonnegative initial conditions for the system above, it is straightforward to show that each of the state variables remains nonnegative for all *t* > 0. Moreover, summing equations (1)–(5), we find that the size of the total population, *N*_*i*_(*t*), satisfies

(6) $$\begin{array}{c} \frac{{\text{d}N}_{i}\left(t\right)}{\text{d}t}\leq \Lambda -\mu N_{i}. \end{array}$$

Integrating the above inequality equation, we find

(7) $$\begin{array}{c} N_{i}\left(t\right)\leq \frac{\Lambda }{\mu }+N_{i}\left(0\right)e^{-\mu t}. \end{array}$$

The result shows the total population size *N*_*i*_(*t*) bounded in this case, and naturally, it follows that each of the compartment states (i.e., *S*, *L*, *M*, *C*, and *R*) are also bounded.

## 3. Estimation of Model Parameters

**Figure 3:**
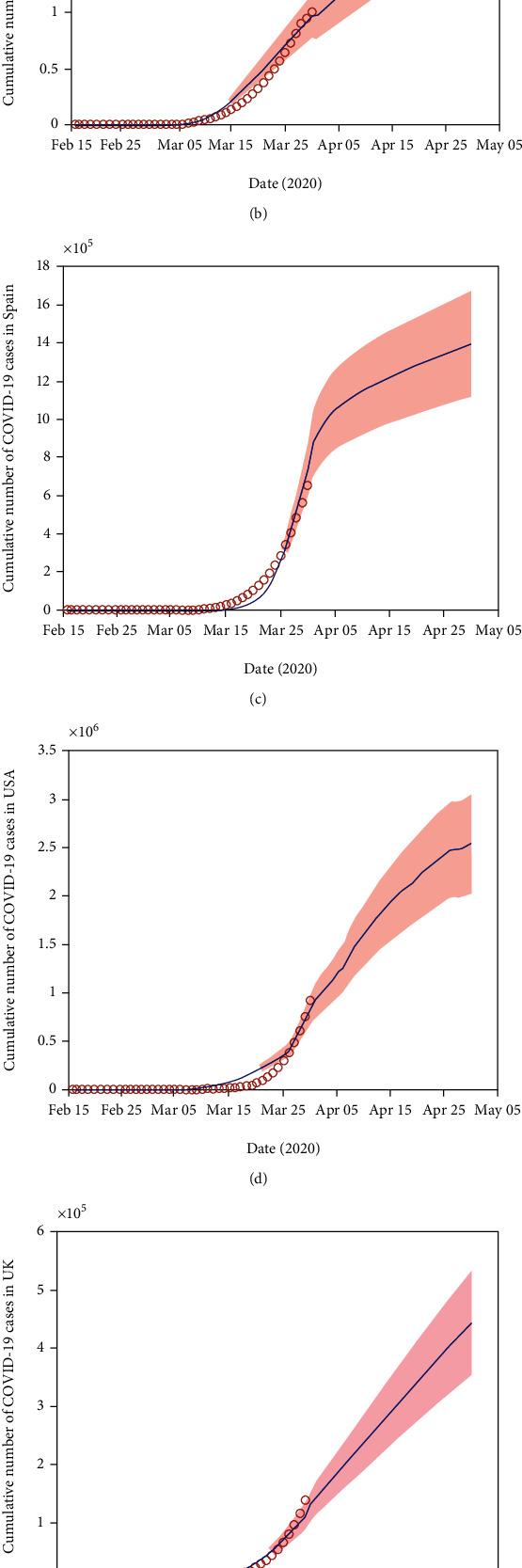
Measured and predicted number of cumulative COVID-19 cases from 15^th^ February to 30^th^ March 2020 (red dot) in six different high-burden countries, (a) Australia, (b) Italy, (c) Spain, (d) the USA, (e) the UK, and (f) Canada, and the corresponding model (the blue-solid curve) with the 95% confidence interval (CI) measure in the rose-shaded limits.

This section estimated the model parameters based on the available six countries' COVID-19 reported case data from http://worldometers.info [1].  Figure 3 presents the curve of cumulative confirmed COVID-19 cases each day from the 15^th^ February to 30^th^ March 2020 in Australia, Italy, Spain, the USA, the UK, and Canada. In order to parameterize the model (1)–(5), we obtained some of the parameter values from the literature (see Table 1). Others were estimated or fitted from the data. We obtained the best-fitted parameter values by minimizing the error using the least-square method between the COVID-19 case data and the solution of the proposed model (1)–(5) (see the blue-solid graph in Figure 3). The model was fitted in MATLAB using the multistart algorithm with 1000 starting points [14]. By keeping the model-convergence results, we estimated the confidence intervals of the model with the normal distribution assumption.

The objective function used in the parameter estimation is as follows:

(8) $$\begin{array}{c} \hat{\theta }=\text{argmin}\overset{n}{\underset{i=1}{\sum }}{\left(\left(\omega_{1}+\omega_{2}\right)L-{\text{data}}_{t_{i}}\right)}^{2}, \end{array}$$

where data_*t*_*i*__ denotes the COVID-19 data and (*ω*_1_ + *ω*_2_)*L* is the corresponding model solution at time *t*_*i*_, while *n* is the number of available actual data points. Findings reveal that the proposed model is well-fitted with the data.

The prediction results from the model are also depicted in Figure 3 to assist in the evidence-based decision-making process. For example, predicted results could assist politicians or decision-makers in the health department of Australia, Italy, Spain, the USA, the UK, and Canada for planning for their health systems' need. They can then implement measures regarding staff resources and hospital beds to meet the challenges of this difficult time. However, if the number of infected individuals follows this trend for the next month, there will be more than 200,000 in Australian, 200,000,000 in Italian, 140,000,000 in Spanish, 250,000,000 in USA, 40,000,000 in UK, and 300,000 in Canadian patients infected by 30^th^ April 2020 as shown in Figure 3.

**Figure 4:**
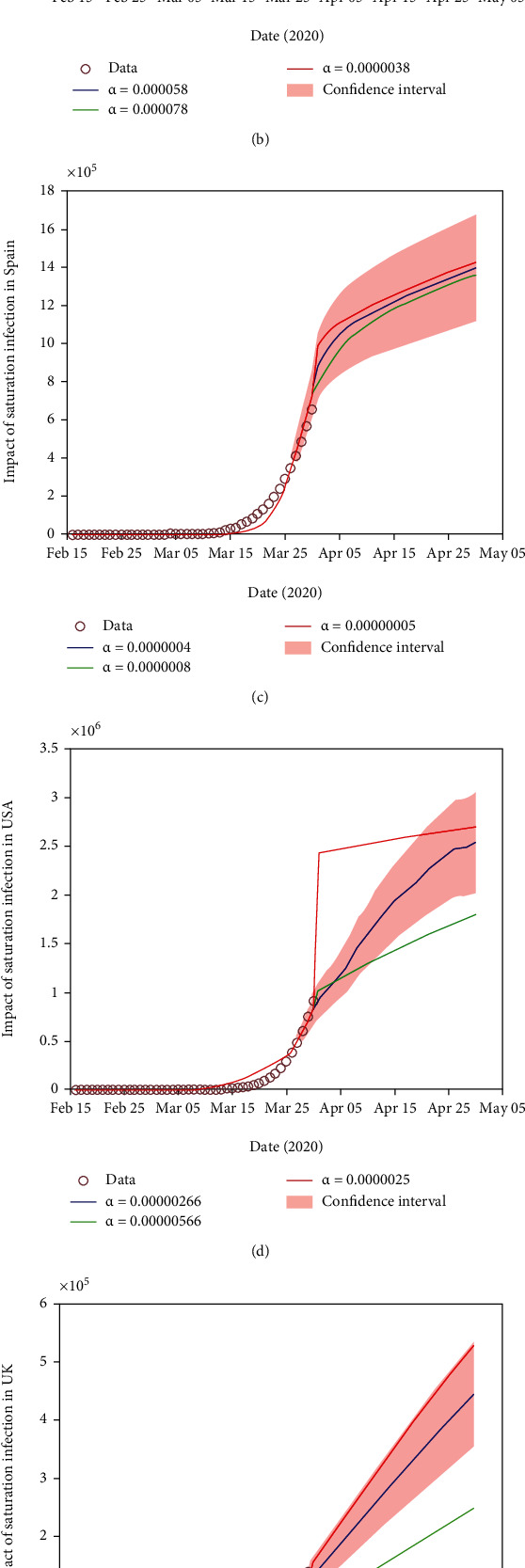
Impact of saturation infection (*α*) on the dynamics of COVID-19 cases in (a) Australia, (b) Italy, (c) Spain, (d) the USA, (e) the UK, and (f) Canada.

Moreover, Figure 4 shows the impact of saturation infection on the dynamics of COVID-19 cases in the six countries Australia, Italy, Spain, the USA, the UK, and Canada. We observed that saturation infection negatively correlates with COVID-19 cases, which means increasing saturation infection will reduce the COVID-19 cases. Further, decreasing saturation infection will increase the COVID-19 cases in all six burden countries.

## 4. Impact of Age Structure on the Dynamics of COVID-19

**Figure 5:**
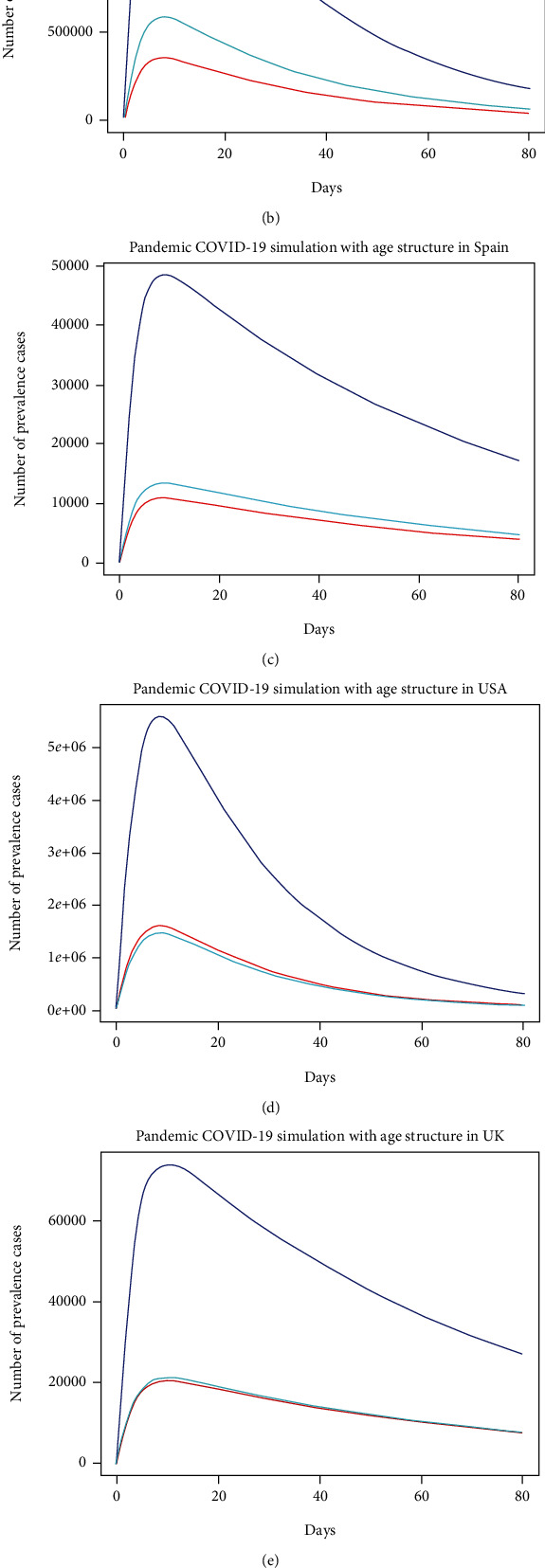
Shows the COVID-19 prevalence in each of the age groups as the epidemic progresses in six countries: (a) Australia, (b) Italy, (c) Spain, (d) the USA, (e) the UK, and (f) Canada.

In this section, we explored the impact of three age classes, including children (0-14 years), adults (15-64 years), and older (over 64 years), on the transmission dynamics of COVID-19 cases over 80 days in the six high-burden countries. In Australia, Italy, Spain, the USA, the UK, and Canada, the percentages of children (0-14 years) among the total population are 18.72%, 13.45%, 15.02%, 18.46%, 17.63%, and 15.99%, respectively. Further, the percentages of adults (15-64 years) among the total population are 35.39%, 64.47%, 66.50%, 64.69%, 63.89%, and 65.03%, respectively. Finally, the percentages of older people (over 64 years) among the total population are 15.88%, 22.08%, 18.49%, 16.85%, 18.48%, and 18.98%, respectively [22]. Results show that adults are more infected than children and the older population in all countries. However, in Italy, Spain, the UK, and Canada, older people are more infected than children. In contrast, children are more infected than older people in Australia and the USA (see Figure 5).

## 5. Sensitivity Analysis

**Figure 6:**
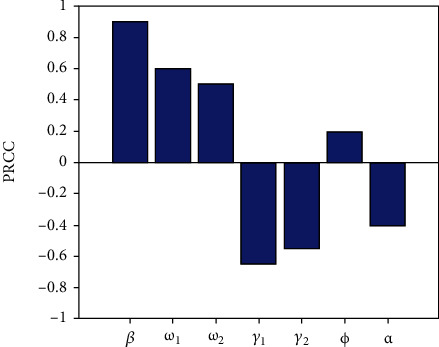
PRCC values depicting the sensitivities of the model output, i.e., the total number of infectious individuals (*M* + *C*) for the estimated parameters *β*,  *ω*_1_,  *ω*_2_,  *γ*_1_,  *γ*_2_,  *ϕ*, and *α*.

**Figure 7:**
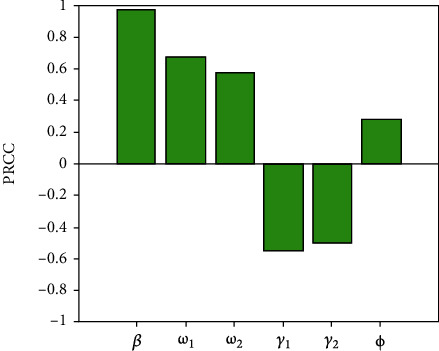
PRCC values depicting the sensitivities of the model output, i.e., the basic reproduction number (*R*_0_) for the estimated parameters *β*,  *ω*_1_,  *ω*_2_,  *γ*_1_,  *γ*_2_, and *ϕ*.

Sensitivity analysis is a handy method to investigate the parameters that most significantly influence on the model outputs [23, 24]. In this study, we performed the partial rank correlation coefficient (PRCC) estimation, a global sensitivity analysis technique proven to be the most reliable and efficient sampling-based method [24, 25]. The analysis conducted about 100,000 simulations by assigning a uniform distribution to each model parameter and using independent sampling. The positive (negative) correlation suggests that a positive (negative) variation in the parameter will increase (decrease) the model outcome [24]. Here, the model outputs we consider are the total number of infectious individuals *M* + *C* (where,  *M* + *C* = (1 + (*γ*_2_(*ϕ* + *γ*_1_ + *μ*) + *ϕω*_1_)/*ω*_1_(*γ*_2_ + *μ*))((*βΛω*_1_^2^(*ω*_1_(*γ*_2_ + *μ*) + *ω*_2_(*ϕ* + *γ*_1_ + *μ*) + *ϕω*_1_) − *ω*_1_*μ*(*γ*_2_ + *μ*)(*ω*_1_ + *ω*_2_ + *μ*)(*ϕ* + *γ*_1_ + *μ*))/(*ω*_1_(*γ*_2_ + *μ*) + *ω*_2_(*ϕ* + *γ*_1_ + *μ*) + *ϕω*_1_)(*ω*_1_ + *ω*_2_ + *μ*)(*ϕ* + *γ*_1_ + *μ*)(*αω*_1_*μ* + *βω*_1_))) and the basic reproduction number *R*_0_(where, *R*_0_ = (*Λ*/*μ*)*βω*_1_/(*ω*_1_ + *ω*_2_ + *μ*)(*γ*_1_ + *ϕ* + *μ*) + (*Λ*/*μ*)*β*(*μω*_2_ + *ω*_1_*ω*_2_ + *ω*_1_*ϕ* + *ω*_2_*ϕ*)/(*γ*_2_ + *μ*)(*ω*_1_ + *ω*_2_ + *μ*)(*γ*_1_ + *ϕ* + *μ*)). The PRCC results of (*M* + *C*) and *R*_0_ corresponding to the model parameters *β*, *ω*_1_, *ω*_2_, *γ*_1_, *γ*_2_, *ϕ*, and *α* are displayed in Figures 6 and 7. Parameters *β*, *ω*_1_, *ω*_2_, and *ϕ* have positive PRCC values, implying that a positive change of these parameters will increase the total number of infectious individuals (*M* + *C*) and the basic reproduction number. In contrast, parameters *γ*_1_, *γ*_2_, and *α* have negative PRCC values, which imply that increasing these parameters will decrease the total number of infectious individuals (*M* + *C*) and *R*_0_.

## 6. Discussion and Concluding Remarks

In this paper, we presented an age-structured SLMCR compartmental model with nonlinear incidence. We estimated the number of cases from COVID-19 infection and applied it to data from the COVID-19 epidemics in Australia, Italy, Spain, the USA, the UK, and Canada to predict the COVID-19 situation until 30^th^ April 2020. After model calibration, we estimated the transmission rates of 4.6214 × 10^−8^, 1.2292 × 10^−8^, 3.3182 × 10^−7^, 3.1828 × 10^−7^, 1.1526 × 10^−7^, and 2.0731 × 10^−7^, respectively, in Australia, Italy, Spain, the USA, the UK, and Canada. The model estimates revealed a strong relationship between the transmission rate and the number of cases in COVID-19 of the selected countries, which is a consistent result with other studies related to COVID-19 [26–28].

Within the six selected countries, we found that Spain has the highest transmission rate than the other countries, which may increase the massive number of COVID-19 cases and make it one of the worst situations in Spain. The Spanish government may not have taken proper action at the initial stage to control transmission, including handwashing, social distancing, and good respiratory hygiene. For instance, in China, they took immediate action for transmission control, including lockdown in every city; that is how they were able to minimize the outbreak of COVID-19. Our finding is consistent with such typical observations because COVID-19 mainly spreads from person to person through droplet transmission. Droplets cannot go through the skin and can only lead to infection if they touch the mouth, nose, or eye. Therefore, it is essential to protect susceptible individuals from COVID-19 exposure from a public health perspective by effectively reducing the contact rate between susceptible and infectious individuals.

Age is a significant risk factor that can increase the severity of the outbreak of COVID-19. In our study, we found that adults are more infected than children and older people. The SARS-CoV-2 may be present for several days before there are any symptoms, and many adult people will have few or no symptoms at all. Surveillance data confirm that the SARS-CoV-2 virus is more in adults and had the highest rates of COVID-19 cases [29]. Modelling studies have found that children are less likely to acquire an infection and are much less likely to show symptoms [30], similar to our results in Italy, Spain, the UK, and Canada but dissimilar in Australia and the USA. This information will assist policymakers in strategy development.

Estimation of transmission rates from different settings must be done with caution, though, as the pattern of a pandemic, the standard of care, and, as a result, the number of cases are time and setting dependent. For instance, very few cases have been reported so far in Bangladesh [31]. In this developing country, the health system is indigent and not comprehensive to cover every citizen, which leads to fewer reported cases. Therefore, data from other countries, in particular, the number of cases by date of COVID-19 onset, is necessary to better understand the variability in cases across different settings.

Our model determined that from the explicit expression for the total number of infectious individuals (*M* + *C*) and the basic reproduction number *R*_0_, it is clear that they depended on transmission rates *β*, progression rates *ω*_1_ and *ω*_2_, recovery rates *γ*_1_ and *γ*_2_, comorbidity rate *ϕ*, and infection saturation rate *α*. From the sensitivity analysis findings, it is also clear that the most critical parameter was the transmission rate, *β*, followed by the recovery rate, *γ*_1_. Therefore, to control and eradicate COVID-19 infection, it is crucial to consider the following strategies: (i) the first and most important strategy is to minimize the contact rates *β* with infected individuals by decreasing the values of *β*; (ii) the second-most important strategy is to increase the recovery rate *γ*_1_ of infective individuals through treatment. Therefore, we suggest that the most feasible and optimal strategy to eliminate COVID-19 in the six different countries, i.e., Australia, Italy, Spain, the USA, the UK, and Canada, is to reduce contact rates as well as increase the treatment rate. Finally, the application of the proposed model and its related outputs can be extended to many other countries dealing with such a critical outbreak of COVID-19 to control this global pandemic disease.

## Figures and Tables

**Table 1:** Depiction and estimation of the model parameters for six countries.

Countries	Parameters	Description	Estimated values	References
Australia	*N*	Population in 2020	25,499,884	[15]
	*μ*	Death rate	$\frac{1}{70}\text{ y}{\text{r}}^{-1}$	[16]
	*β*	Transmission rate	4.6214 × 10^−8^	Fitted
	*ω*_1_	Progression rate from *L* to *M*	0.001	Assumed
	*ω*_2_	Progression rate from *L* to *C*	0.001	Assumed
	*γ*_1_	Recovery rate from *M* to *R*	0.1	Assumed
	*γ*_2_	Recovery rate from *C* to *R*	0.1	Assumed
	*α*	Infection saturation rate	0.00001	Assumed
	*ϕ*	Comorbidity rate	0.3	Assumed
	*Λ*	Recruitment rate	1	

Italy	*N*	Population in 2020	60,461,826	[17]
	*μ*	Death rate	$\frac{1}{70}\text{ y}{\text{r}}^{-1}$	[16]
	*β*	Transmission rate	1.2292 × 10^−8^	Fitted
	*ω*_1_	Progression rate from *L* to *M*	0.009	Assumed
	*ω*_2_	Progression rate from *L* to *C*	0.009	Assumed
	*γ*_1_	Recovery rate from *M* to *R*	0.14	Assumed
	*γ*_2_	Recovery rate from *M* to *R*	0.10	Assumed
	*α*	Infection saturation rate	0.0000058	Assumed
	*ϕ*	Comorbidity rate	0.3	Assumed
	*Λ*	Recruitment rate	1	

Spain	*N*	Population in 2020	46,754,778	[18]
	*μ*	Death rate	$\frac{1}{70}\text{ y}{\text{r}}^{-1}$	[16]
	*β*	Transmission rate	3.3182 × 10^−7^	Fitted
	*ω*_1_	Progression rate from *L* to *M*	0.00034	Assumed
	*ω*_2_	Progression rate from *L* to *C*	0.00034	Assumed
	*γ*_1_	Recovery rate from *M* to *R*	0.3	Assumed
	*γ*_2_	Recovery rate from *M* to *R*	0.3	Assumed
	*α*	Infection saturation rate	0.0000004	Assumed
	*ϕ*	Comorbidity rate	0.3	Assumed
	*Λ*	Recruitment rate	1	

USA	*N*	Population in 2020	331,002,651	[19]
	*μ*	Death rate	$\frac{1}{70}\text{ y}{\text{r}}^{-1}$	[16]
	*β*	Transmission rate	3.1828 × 10^−7^	Fitted
	*ω*_1_	Progression rate from *L* to *M*	0.02	Assumed
	*ω*_2_	Progression rate from *L* to *C*	0.01	Assumed
	*γ*_1_	Recovery rate from *M* to *R*	0.02	Assumed
	*γ*_2_	Recovery rate from *C* to *R*	0.008	Assumed
	*α*	Infection saturation rate	0.00000266	Assumed
	*ϕ*	Comorbidity rate	0.3	Assumed
	*Λ*	Recruitment rate	1	

UK	*N*	Population in 2020	67,886,011	[20]
	*μ*	Death rate	$\frac{1}{70}\text{ y}{\text{r}}^{-1}$	[16]
	*β*	Transmission rate	1.1526 × 10^−7^	Fitted
	*ω*_1_	Progression rate from *L* to *M*	0.0003	Assumed
	*ω*_2_	Progression rate from *L* to *C*	0.0003	Assumed
	*γ*_1_	Recovery rate from *M* to *R*	0.1	Assumed
	*γ*_2_	Recovery rate from *C* to *R*	0.02	Assumed
	*α*	Infection saturation rate	0.00001	Assumed
	*ϕ*	Comorbidity rate	0.3	Assumed
	*Λ*	Recruitment rate	1	

Canada	*N*	Population in 2020	37,742,154	[21]
	*μ*	Death rate	$\frac{1}{70}\text{ y}{\text{r}}^{-1}$	[16]
	*β*	Transmission rate	2.0731 × 10^−7^	Fitted
	*ω*_1_	Progression rate from *L* to *M*	0.0003	Assumed
	*ω*_2_	Progression rate from *L* to *C*	0.0003	Assumed
	*γ*_1_	Recovery rate from *M* to *R*	0.2	Assumed
	*γ*_2_	Recovery rate from *C* to *R*	0.2	Assumed
	*α*	Infection saturation rate	0.00001	Assumed
	*ϕ*	Comorbidity rate	0.3	Assumed
	*Λ*	Recruitment rate	1	

## Data Availability

All data will be available upon request.
